# High-intensity-focused ultrasound treatment for the chronic venous disease based on the Cure Conservatrice et Hémodynamique de l’Insuffisance Veineuse en Ambulatoire (CHIVA) strategy

**DOI:** 10.1016/j.jvsv.2025.102233

**Published:** 2025-03-19

**Authors:** Luis Miguel Izquierdo Lamoca, Teresa Reyero Postigo, Sonia Morán Escalona, Juan Francisco Giráldez Arranz, Ana Aguinaco Acosta

**Affiliations:** a Angiology, Vascular and Endovascular Surgery Service, Instituto Vascular Internacional, Hospital Universitario Madrid Montepríncipe, Madrid, Spain; b Department of Clinical Sciences, Universidad CEU-San Pablo School of Medicine, Madrid, Spain; c HM Camilo Jose Cela University, School of Medicine, Madrid, Spain

**Keywords:** CHIVA, Chronic venous disease, High-intensity focused ultrasound, Thermal ablation, Varicose veins

## Abstract

**Objective:**

Chronic venous disease management has significantly advanced with minimally invasive techniques like endovenous thermal ablation. High-intensity focused ultrasound (HIFU) is a noninvasive alternative thermal ablation method enabling targeted vein closure without percutaneous access. This study evaluates the efficacy of HIFU treatment, combined with the Cure Conservatrice et Hémodynamique de l'Insuffisance Veineuse en Ambulatoire (CHIVA) strategy, in occluding leak points in patients with superficial venous reflux.

**Methods:**

This retrospective study included patients treated for chronic venous disease using the SONOVEIN device from March 2020 to February 2024. Inclusion criteria were symptomatic patients (CEAP ≥C2) with ultrasound-confirmed truncal reflux. Patients under 18 years, with <12 months life expectancy, or isolated venous flow obstruction were excluded. Treatments followed CHIVA principles, targeting leak points and proximal vein segments for occlusion. Primary endpoints were leak point occlusion rates and procedural safety. In this study, primary efficacy was defined as vein closure with one treatment, whereas secondary efficacy was defined as the need for more.

**Results:**

A total of 204 limbs in 183 patients (131 females, 52 males; mean age, 55.2 years) underwent HIFU treatment. Primary leak point occlusion rate at 1 week, and 1, 6, 12, and 24 months were 85.1 %, 91.8%, 93.7%, 94.3%, and 95.5%, respectively. Secondary closure rates at 1 week, and 1, 6, 12, and 24 months were 89.6 %, 95.4%, 95.0%, 95.4%, and 95.7%, respectively. Overall primary and secondary cumulative closure rates at 24 months were 88.1% (95% confidence interval, 77.7%-98.4%), and 92.1% (95% confidence interval, 83.3%-100%), respectively. No major complications were observed.

**Conclusions:**

HIFU achieves high leak point occlusion rates when applied under CHIVA principles, supporting its role as an effective and safe noninvasive alternative for chronic venous disease treatment. The technique minimizes risks associated with thermal ablation, such as skin burns and nerve damage, while addressing limitations of conventional methods. These results highlight the potential of HIFU as a disruptive technology in venous disease management. Further studies should assess its long-term efficacy and safety.

**Article Highlights**

• **Type of Research:** Single-center, non-randomized, retrospective study
• **Key Findings:** Use of high-intensity focused ultrasound (HIFU) a complete extracorporeal thermal ablation in the Cure Conservatrice et Hémodynamique de l'Insuffisance Veineuse en Ambulatoire (CHIVA) strategy to close venous leak points in 204 limbs with superficial venous reflux obtains a primary and secondary leak point occlusion rate at 24 months of 95.5% and 95.7%, respectively, with no major complications.
• **Take Home Message:** HIFU is an effective and safe noninvasive alternative for chronic venous insufficiency treatment to open surgery and endothermal ablation techniques. These results highlight HIFU’s potential as a disruptive technology in venous disease management.

The management of chronic venous disease (CVD) experienced significant advancements with the development of minimally invasive techniques. Endovenous thermal ablation techniques (EVTA), mainly endovenous laser therapy (EVLT) and radiofrequency ablation, have become standard treatments.^1^^,^^2^

Although these techniques are effective, they have certain limitations, particularly in terms of patient eligibility (eg, tortuous veins, small diameter, perforating veins) and associated benefits for patients (eg, days off work, adverse events). In this context, high-intensity focused ultrasound (HIFU) emerges as a promising thermal ablation technique due to its ability to perform tissue coagulation noninvasively. The development of HIFU has broadened the role of ultrasound (US) from diagnostic to therapeutic interventions.

HIFU works by focusing US pulses at a specific target, generating both thermal and mechanical effects. The thermal effects lead to protein denaturation, coagulative necrosis, and cell death that triggers hyalinization and inflammation of the adventitia and surrounding adipose tissue, leading to progressive fibrotic remodeling process. This technology has been applied in various fields of medicine, particularly in oncology, where it is used both for direct tumor ablation and as an adjunct to optimize existing therapies (breast, thyroid, liver, and prostate cancers). Additionally, HIFU has applications in ophthalmology and in the ablation of basal ganglia to manage hyperkinetic disorders, such as Parkinson’s disease.^3^^,^^4^

By precisely focusing US energy, HIFU can induce targeted thermal vein closure. As an extracorporeal treatment, it offers the potential to close veins similarly to EVTA but without the technical and anatomic limitations inherent to that approach. Although its use in CVD has been primarily experimental, recent years have seen growing evidence, including *in vivo* studies and clinical trials, supporting the application of HIFU for treating varicose veins.^5^, ^6^, ^7^, ^8^

The Cure Conservatrice et Hémodynamique de l’Insuffisance Veineuse en Ambulatoire (CHIVA) strategy has established itself as a conservative alternative in the management of chronic venous insufficiency. This technique focuses on the preservation of the superficial venous system by correcting the pathologic hemodynamic circuits, with the leak point closure to eliminate reflux as the key therapeutic maneuver, instead of radical ablation of the affected vein.^9^, ^10^, ^11^ When combined with the CHIVA technique, HIFU offers a promising new approach in the treatment of CVD.

This study aims to assess both the leak point closure, clinical effectiveness, and safety of HIFU for treating CVD in patients with superficial venous reflux based on the CHIVA strategy. Here, we present the procedure description and our 24-month results.

## Methods

This study is a non-randomized, retrospective cohort analysis. The Strengthening the Reporting of Observational Studies in Epidemiolog (STROBE) guidelines and checklist for cohort studies were used as reporting standard recommendations.^12^ Medical records of patients treated for CVD using a robotic HIFU device (SONOVEIN, Theraclion) were collected from the Instituto Vascular Internacional at Vascular and Endovascular Surgery Service of HM Montepríncipe Hospital (Madrid, Spain) from March 2020 to February 2024. The data analyzed included demographics, Clinical, Etiological, Anatomical, and Pathophysiological (CEAP) clinical classification, revised Venous Clinical Severity Score (rVCSS), treatment indication and characteristics, clinical outcomes, treated veins patency, presence of reflux, and complications. This study was conducted with the prior approval of the hospital’s ethics and research committee (CEIm HM Hospitals code 24.02.2297-GHM).

### Inclusion criteria

Patients included in the study had to be symptomatic and classified as CEAP C ≥2 and with Doppler ultrasound (DUS)-identified pathologic vein reflux lasting >0.5 seconds, in great saphenous vein (GSV), accessory anterior saphenous vein (AASV), and/or small saphenous vein (SSV).^2^ All veins incorporated into the study were included, irrespective of their diameter. We excluded perforator veins and neovascularization stumps from the analysis. Only patients under 18 years of age, with a life expectancy of less than 12 months, or with venous flow obstruction as only component of their CVD were excluded from the study.

### Pre-procedure vascular study

Pre-procedure study work-up included anamnesis; physical examination in the standing position; rVCSS; CEAP score evaluation; and abdominal and lower limbs DUS, B-mode, color, and Doppler echography to identify superficial and/or deep reflux and collateral pathways and their diameters, creating a venous map, to determine the veins to be treated. No preanesthetic or preoperative test were performed as in conventional varicose vein surgery procedures. Patients did not need to fast nor stop their usual medications, including anticoagulants.

### Device and procedure description

**Fig 1:**
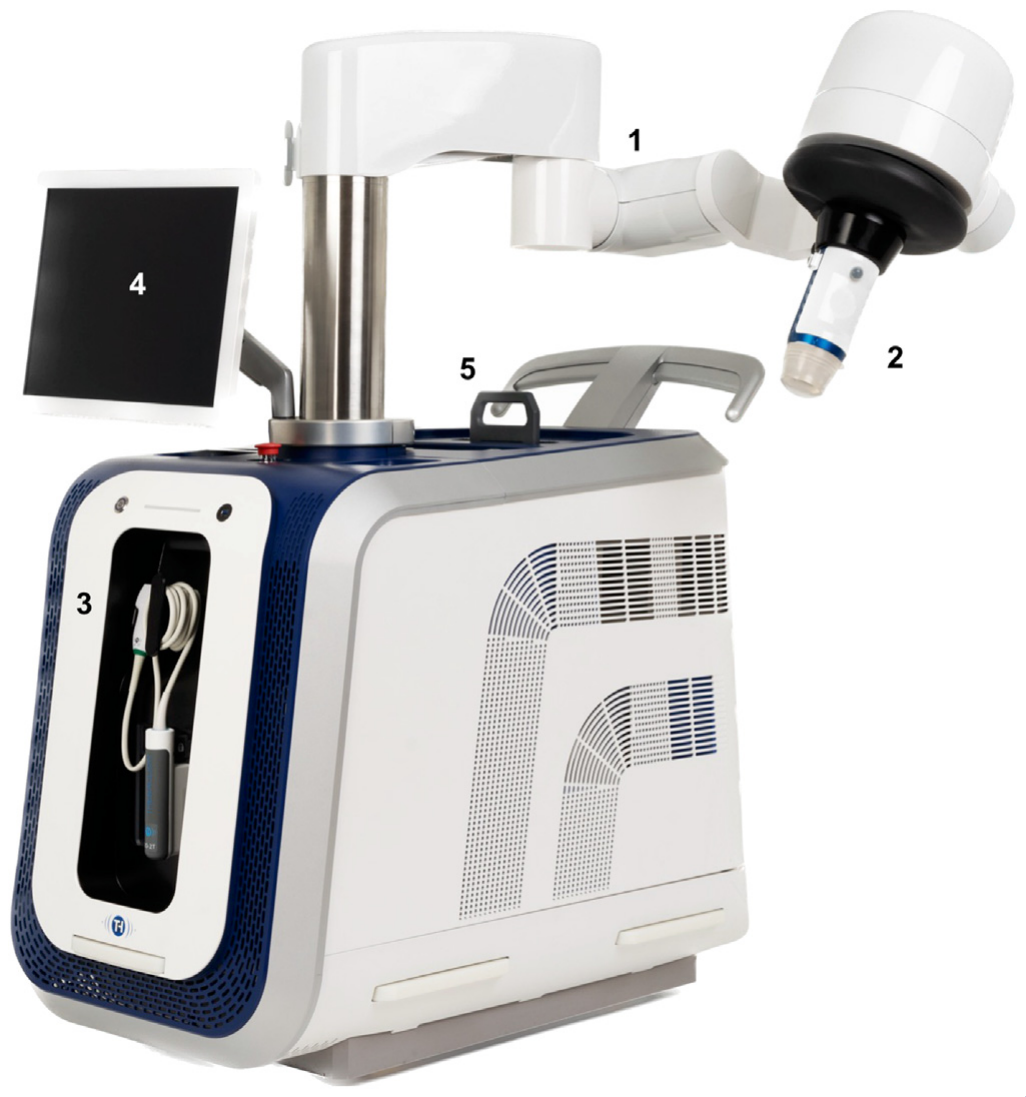
SONOVEIN HD version device. (1) Robotic arm. (2) Visualization and treatment unit (VTU). (3) Ten-MHz linear array ultrasound (US) handheld probe. (4) Touch screen control console. (5) Cooling system (courtesy of Theraclion).

**Fig 2:**
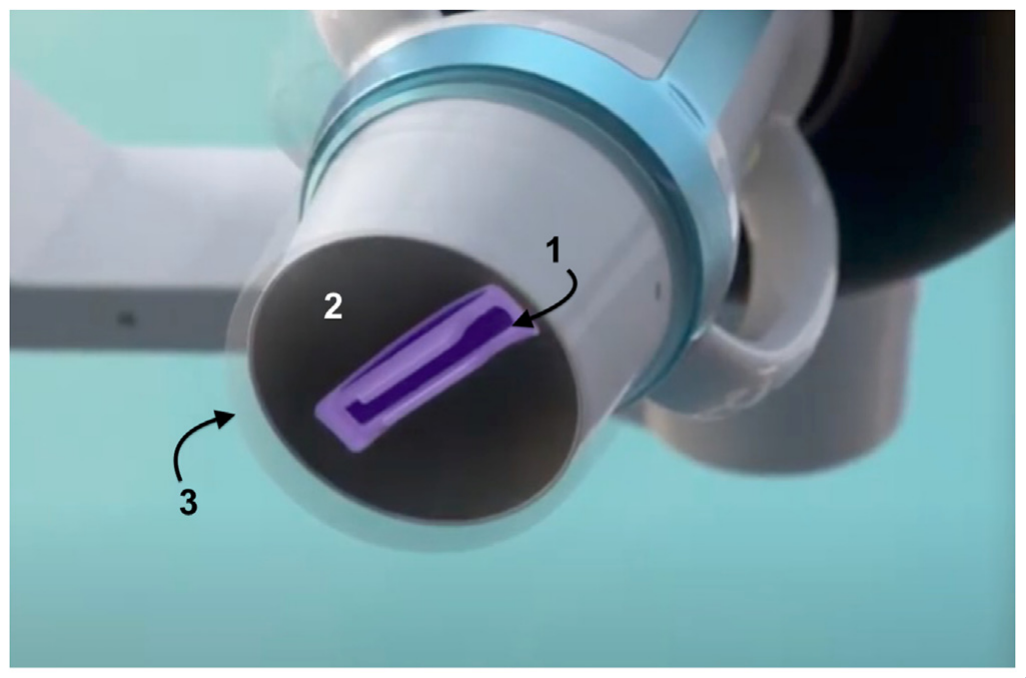
SONOVEIN HD. Visualization and treatment unit (VTU) detail (1) Linear array ultrasound probe (10 MHz). (2) Piezoelectric dome-shaped transducer. (3) Single-use balloon-shaped membrane cooling system (courtesy of Theraclion).

Treatments were performed on an outpatient basis in a clean clinical room using the SONOVEIN device (Theraclion), which features a robotic arm with a visualization and treatment unit (VTU) and a touchscreen for live monitoring. The VTU includes a 10-MHz linear imaging probe for automated alignment of the target vein and a piezoelectric dome-shaped transducer for HIFU delivery (Figs 1 and 2). A single-used coupling balloon filled with degassed liquid was used to ensure effective acoustic coupling between the VTU and the treatment area. The liquid was cooled down to 10 °C during the entire procedure to prevent skin burns.

During the study, three versions of the device were used. The SONOVEIN 1, used from March 2020 to December 2021, was limited to 4- and 8-second pulses (which involve 30 to 60 seconds pause time between pulses), whereas the next generation of SONOVEIN S was used from December 2021 to December 2022, and SONOVEIN HD was used from December 2022 to the end of the study; these also have 0.5- and 1-second pulses available, allowing faster treatment with 10 seconds pause time.

Before the procedure, the treatment area was mapped on the patient’s skin, and the diameter of the target vein was measured in standing position. Small doses of 1% mepivacaine (<10 mL) were injected under US guidance around the target veins. Patients were positioned supine, with the treated leg immobilized using a vacuum cushion.

The VTU was first positioned manually on the proximal part of the treatment area, then robotically adjusted to position the focus on the vein wall. An ablation sequence is defined by HIFU pulses delivered across the transverse axis of the vein. This sequence is repeated along the longitudinal vein axis, every 3 mm.

**Fig 3:**
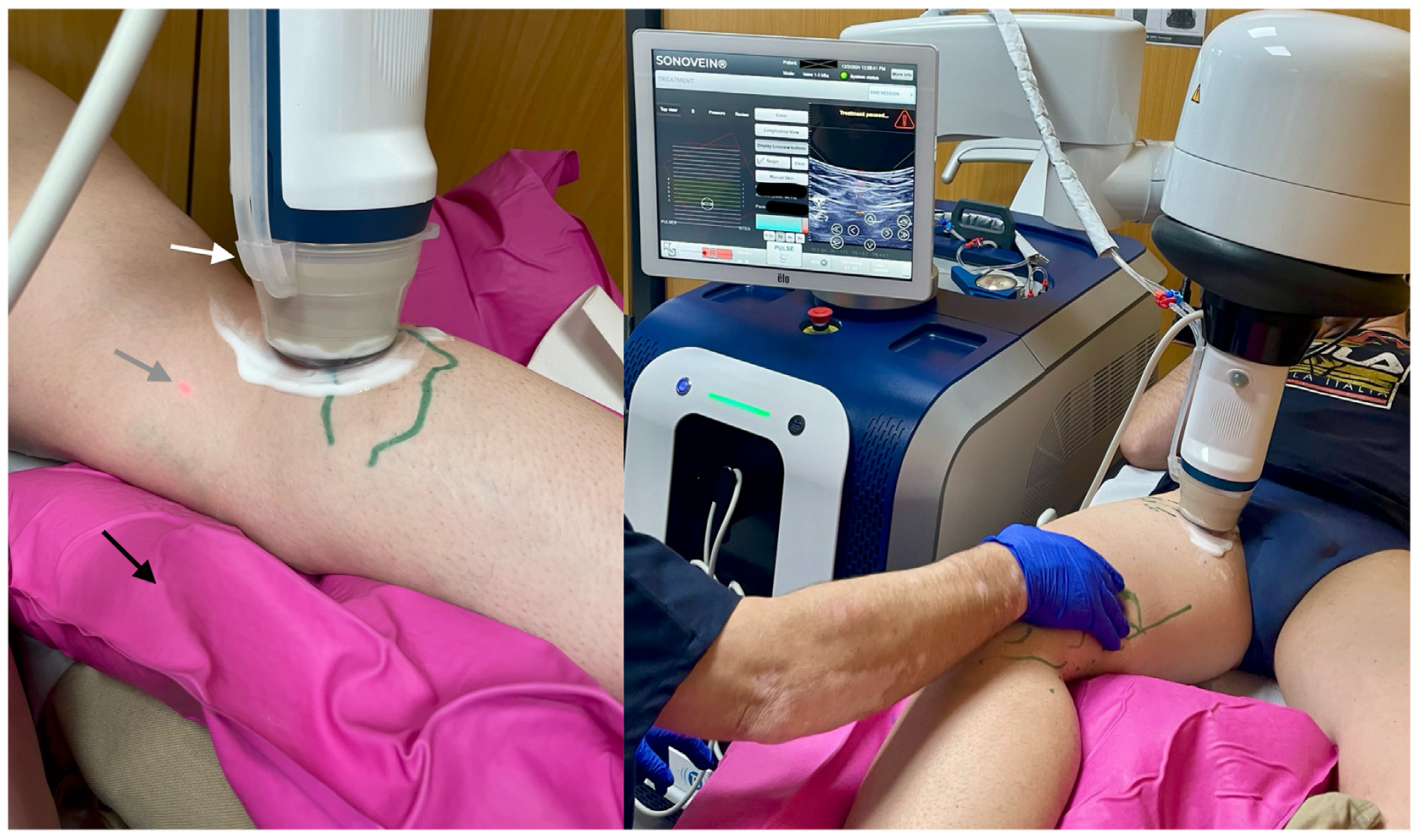
SONOVEIN treatment set-up. *Left*, visualization and treatment unit (VTU) detail. *White arrow* points to VTU balloon-shaped membrane cooling system in contact with patient skin. *Gray arrow* to safety laser system light. *Black arrow* to vacuum cushion. *Right general view*, position the patient with the leg immobilized on the vacuum cushion in slight Trendelenburg to empty the vein; the operator’s hand on the leg allows to perform distal compressions of the limb to better visualize the vein.

The corresponding acoustic power and the number of pulses depend on target depth and diameter, respectively. Between two consecutive pulses, the cooling time was automatically set by the device software to allow the skin to cool down. For safety reasons, two features are also integrated: a laser detection system that pauses the treatment if patient movement is detected during pulses (Fig 3), and a warning from the device software when the vein to be treated is less than 8 mm from the skin. To overcome this limitation, a saline or anesthetic swab could be used to obtain a greater distance between the skin and the target vein.

**Fig 4:**
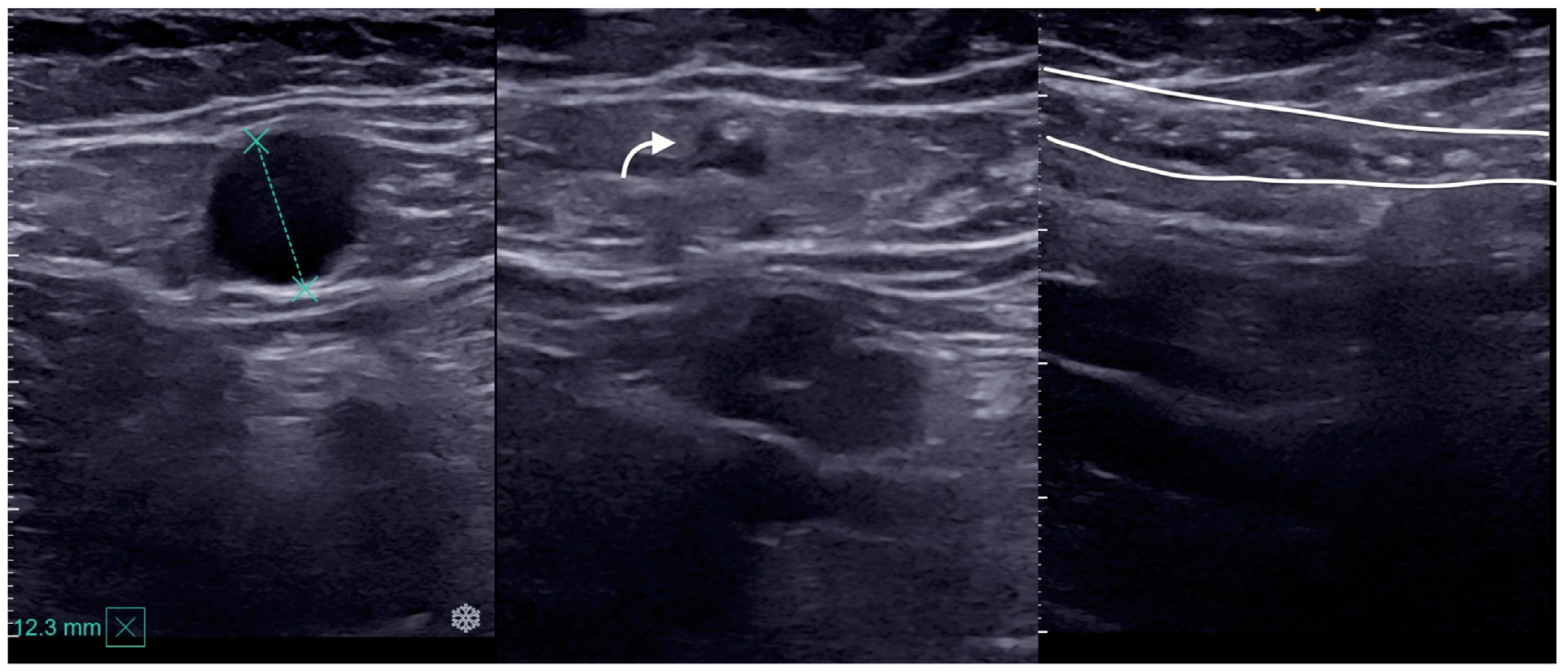
Post-procedure ultrasound images. *Left*, pre procedure axial view of a 12-mm great saphenous vein (GSV). *Middle*, axial view, GSV damaged and retracted immediately after treatment (*arrow*). *Right*, longitudinal view. *White lines* delineate the damaged vein.

In our protocol following the CHIVA principles, the leak point was treated in the same way as when applying laser. A segment of vein close to the leakage point of around 5 cm in the secondary network (N2) was treated: GSV, AASV, and SSV, to induce retraction or occlusion of the vessel and/or disappearance of the reflux (Fig 4).

Treating the draining point from a varicose bundle of the tertiary network (N3) is part of the CHIVA strategy. N3 tributaries were treated by HIFU, at their entry point to the main N2 axis if this was more than 8 mm away from the skin, and the rest of the varicose bundle and those at less than 8 mm by foam sclerotherapy (1% polidocanol foam), with the Varixio foam maker device (VB Devices), in the same procedure or delayed in a second session after the HIFU therapy. The so-called N4 and N3 bundles that connect two axial veins of the secondary network, GSV to SSV, for example, must be eliminated, so in these cases, they were closed with HIFU or foam or both in the procedure.

### Post-procedure treatment

Immediately after treatment, patients were allowed to return to their normal activities, avoiding strenuous exercise, and were asked to wear class II compression stockings for 3 to 4 days. According to the prophylactic anticoagulation protocol implemented at our institution following EVLT procedures, thromboprophylaxis was individualized according to patient-specific risk factors, including age, body mass index, prior history of venous thromboembolism, thrombophilia, and immobility.^2^ Patients received rivaroxaban orally or bemiparin subcutaneously, with the dosage adjusted based on the patient’s weight and thrombotic risk. In low-risk patients, thromboprophylaxis was provided only during the peri-procedure period (24-48 hours). For high-risk patients, treatment was extended to 10 days post-procedure.

### Follow-up

Patient follow-ups were scheduled at 1 week, and 1, 6 and 12 months, and then annually.

**Fig 5:**
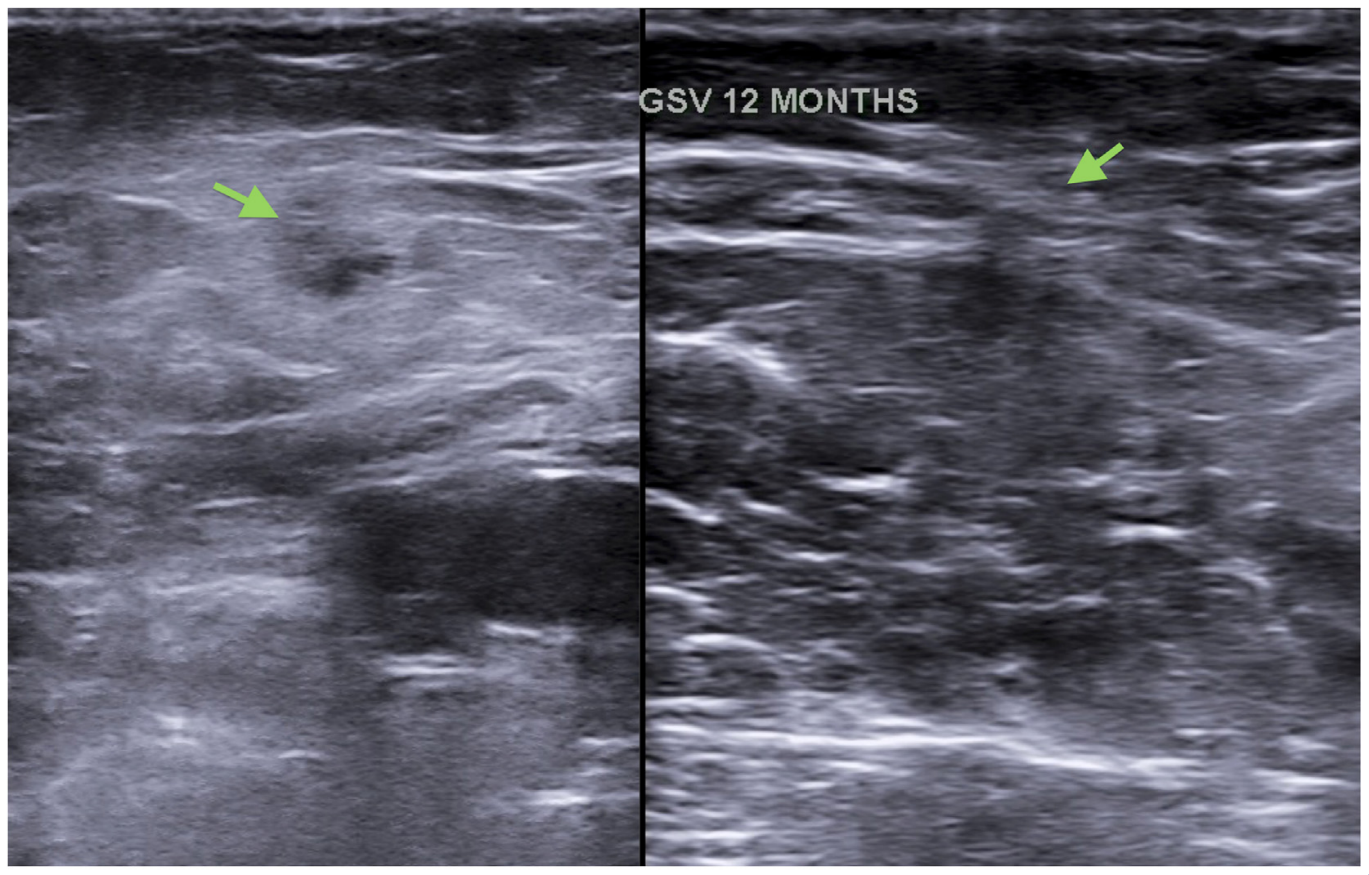
Follow-up ultrasound images: *Left*, 1-month follow-up. Great saphenous vein (GSV) completely occluded (*arrow*). *Right*, GSV almost invisible 12 months after the procedure (*arrow*).

Occlusion and reflux status based on DUS, CEAP class, and rVCSS evaluations were performed at each visit, and adverse events were recorded (Fig 5). The last determination was used in the outcome analysis, as well as the final evaluation comparing vein patency and clinical status before and after the procedure.

The primary efficacy was considered to be the achievement of vein closure with a single treatment, whereas the secondary efficacy was defined as requiring more than one treatment.

### Statistical analysis

All data were collected in an Excel (Microsoft) system database and analyzed using the SPSS statistical program (SPSS Software Inc). Categorical variables are expressed as frequencies and percentages, and continuous variables are presented as mean ± standard deviation. Kaplan-Meier analysis was used to evaluate the leak point closure rate of each follow-up. Categorical variables were analyzed by Fisher exact test and nonparametric χ^2^ test, and the *t*-test or nonparametric Wilcoxon rank test for continuous variables.

## Results

**Fig 6:**
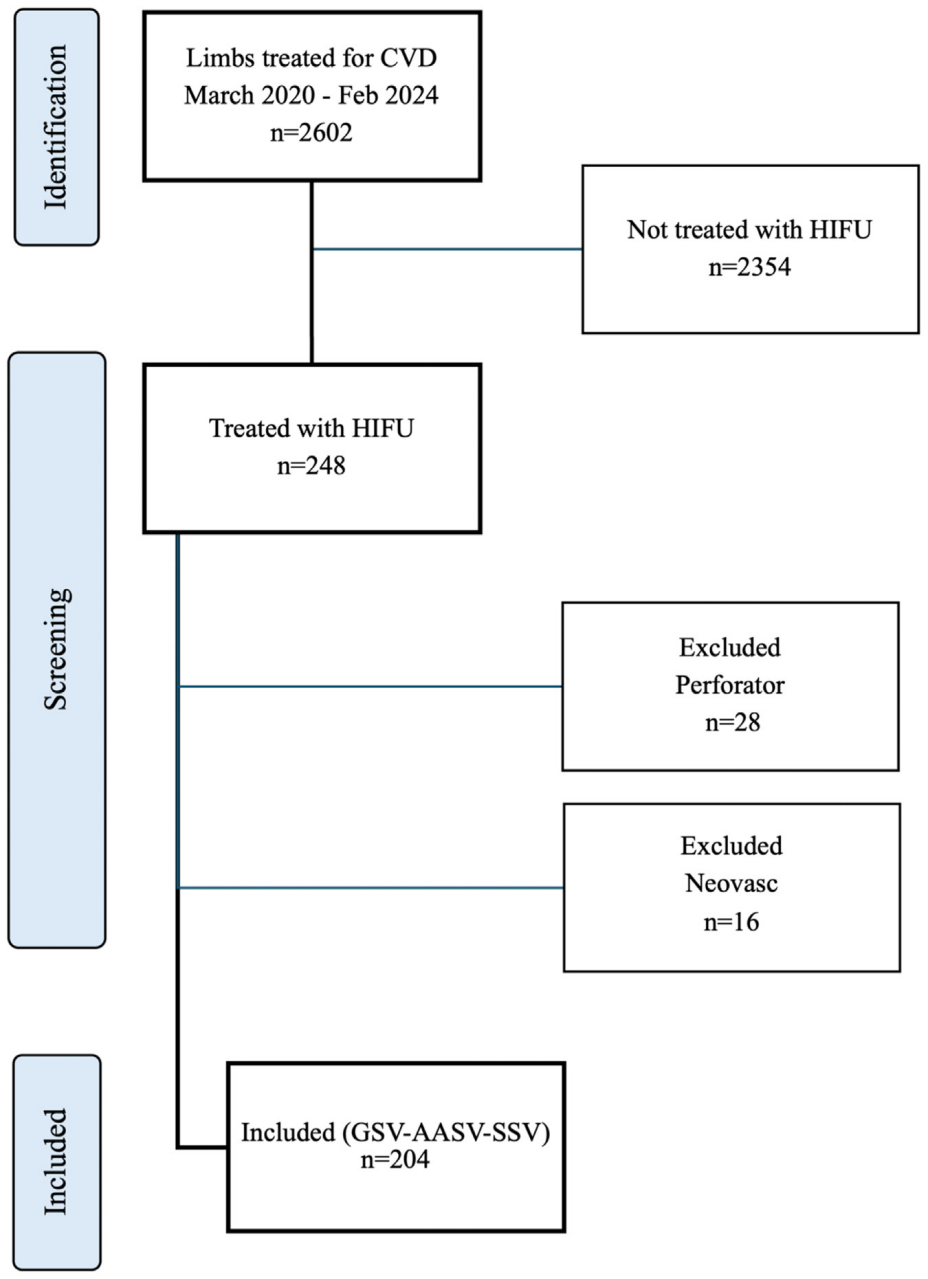
Strengthening the Reporting of Observational Studies in Epidemiology (STROBE) flow chart of the patient selection during the study period, March 2020 to February 2024. *AASV*, Accessory anterior saphenous vein; *CVD*, chronic venous disease; *GSV*, great saphenous vein; *HIFU*, high-intensity focused ultrasound; *SSV*, small saphenous vein.

The study includes 204 limbs in 183 patients. Mean age was 55.2 ± 13.2 years (range, 21-87 years) (Fig 6). Female gender was dominant (71.6% vs 28.4%). CEAP clinical score was class 2 in 15 limbs (7.4%), class 3 in 124 limbs (61.1%), class 4 in 56 limbs (27.6%), class 5 in 2 limbs (1.0%), and class 6 in 6 limbs (3.0%). Median rVCSS score was 6.9 (range, 3-23).

Patients wearing compression prior to intervention were 59 (28.9%) and post-intervention 78 (38.2%).

All procedures (100%) were conducted in a designated clean clinical room in an ambulatory setting.

GSV was the target vein in 152 limbs (74.5%). AASV in 28 (13.7%), and SSV in 24 (11.8%). Vein characteristics are shown in Table I. Local anesthesia was used in 200 patients (98%).

**Table I:** Anatomical distribution and morphologic characteristics of the treated veins

Vein type	Limbs (n = 204)	%	Mean diameter, mm	Mean length, mm
GSV	152	74.5%	7.9 (3-20)	4.8 (1.9-8)
AASV	28	13.7%	6.6 (4-12)	3.7 (1.2-7.2)
SSV	24	11.8%	6.6 (4-12)	2.8 (1-5.3)

*AASV,* Accessory anterior saphenous vein; *GSV,* great saphenous vein; *SSV,* small saphenous vein.

Mean time of SONOVEIN device use from first to last pulse was 33 minutes (range, 6-68 minutes) for SONOVEIN 1, and 23 minutes (range, 4-64 minutes) for SONOVEIN S and HD.

Mean follow-up was 14 months (range, 1-50 months).

Primary leak point closure rate for the series at 1 week, and 1, 6, 12, and 24 months were 85.1 %, 91.8%, 93.7%, 94.3%, and 95.5%, respectively. The results per vein type are shown in Table II.

**Table II:** Primary occlusion rate per vein type over time

	GSV (n = 152)	AASV (n = 28)	SSV (n = 24)
At 1 week			
No. controlled limbs	150	28	24
Occlusion rate, %	82.7	89.3	95.8
At 1 month			
No. controlled limbs	145	27	22
Occlusion rate, %	90.3	96.3	95.7
At 6 months			
No. controlled limbs	119	23	17
Occlusion rate, %	92.4	100	94.1
At 12 months			
No. controlled limbs	80	17	9
Occlusion rate, %	92.5	100	100
At 24 months			
No. controlled limbs	32	6	63
Occlusion rate, %	93.8	100	100

*AASV,* Accessory anterior saphenous vein; *GSV,* great saphenous vein; *SSV,* small saphenous vein.

The secondary closure rate for the series at 1 week, and 1, 6, 12, and 24 months were 89.6%, 95.4%, 95.0%, 95.4%, and 95.7%, respectively (Table III).

**Table III:** Secondary occlusion rate per vein type over time

	GSV (n = 152)	AASV (n = 28)	SSV (n = 24)
At 1 week			
No. controlled limbs	150	28	24
Occlusion rate, %	88.7	89.3	95.8
At 1 month			
No. controlled limbs	146	27	23
Occlusion rate, %	95.2	96.3	95.7
At 6 months			
No. controlled limbs	120	23	16
Occlusion rate, %	94.2	100	94.1
At 12 months			
No. controlled limbs	82	17	9
Occlusion rate, %	93.9	100	100
At 24 months			
No. controlled limbs	34	7	6
Occlusion rate, %	94.1	100	100

*AASV,* Accessory anterior saphenous vein; *GSV,* great saphenous vein; *SSV,* small saphenous vein.

**Fig 7:**
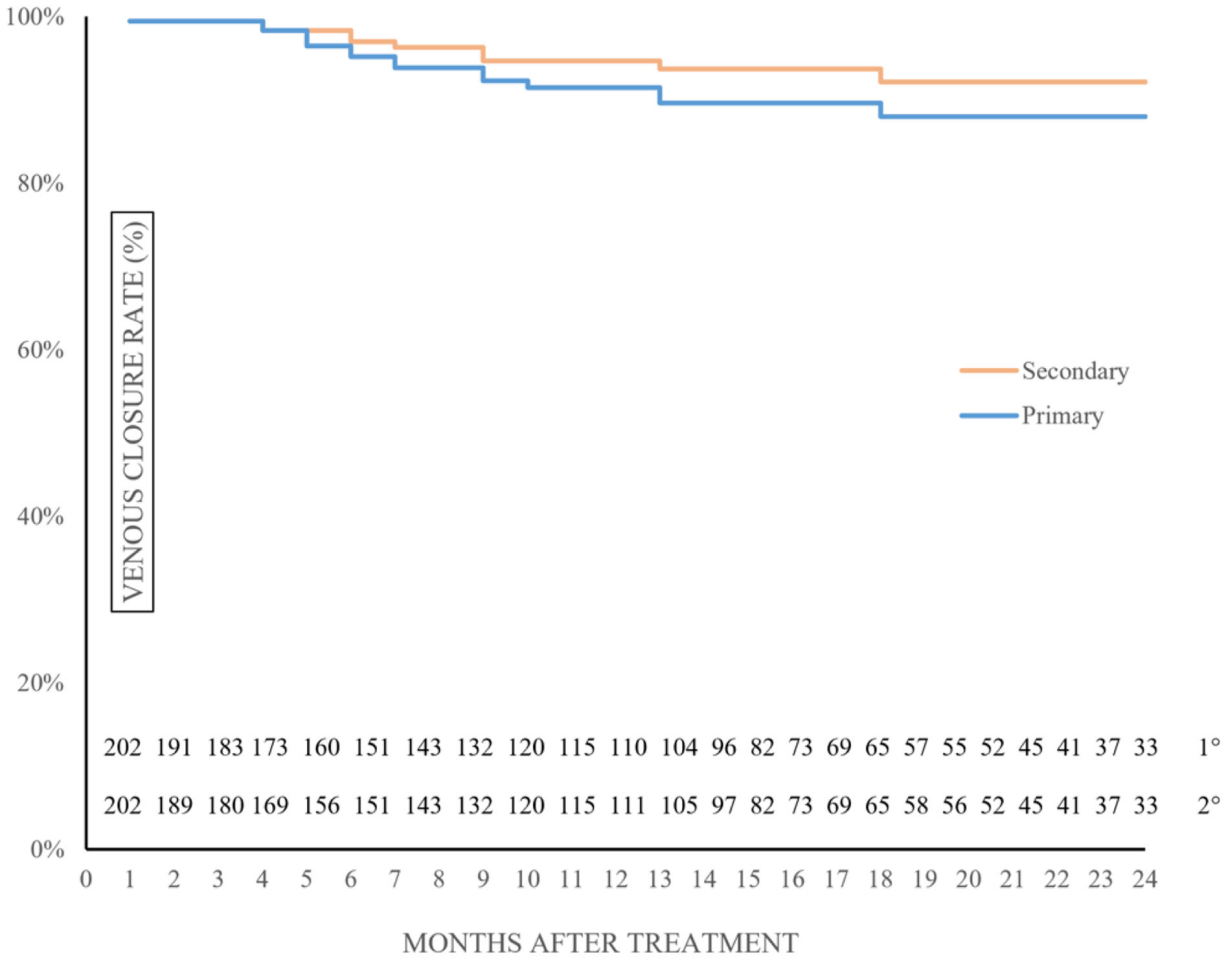
Kaplan-Meier curves for cumulative primary and secondary closure rates. The lower numbers represent limbs at risk at each time interval (all standard error of the mean <10%).

Overall primary and secondary cumulative closure rates at 24 months were 88.1% (95% confidence interval, 77.7%-98.4%) and 92.1% (95% confidence interval, 83.3%-100%), respectively (Fig 7).

Follow-up ultrasound confirmed that only the segment treated with HIFU was occluded; the rest of the treated venous axis remained patent. Reflux disappeared in 96.5% of veins treated; it also disappeared in three of the veins still open after HIFU procedure.

Regarding the N3 network entry points, 17 extremities (8.3%) were treated with HIFU alone, 23 (11.2%) with HIFU and foam, and 164 (80.4%) with foam exclusively. Complementary foam sclerotherapy to varicose bundles in the follow-up was necessary in 32 patients (15.7%)

**Fig 8:**
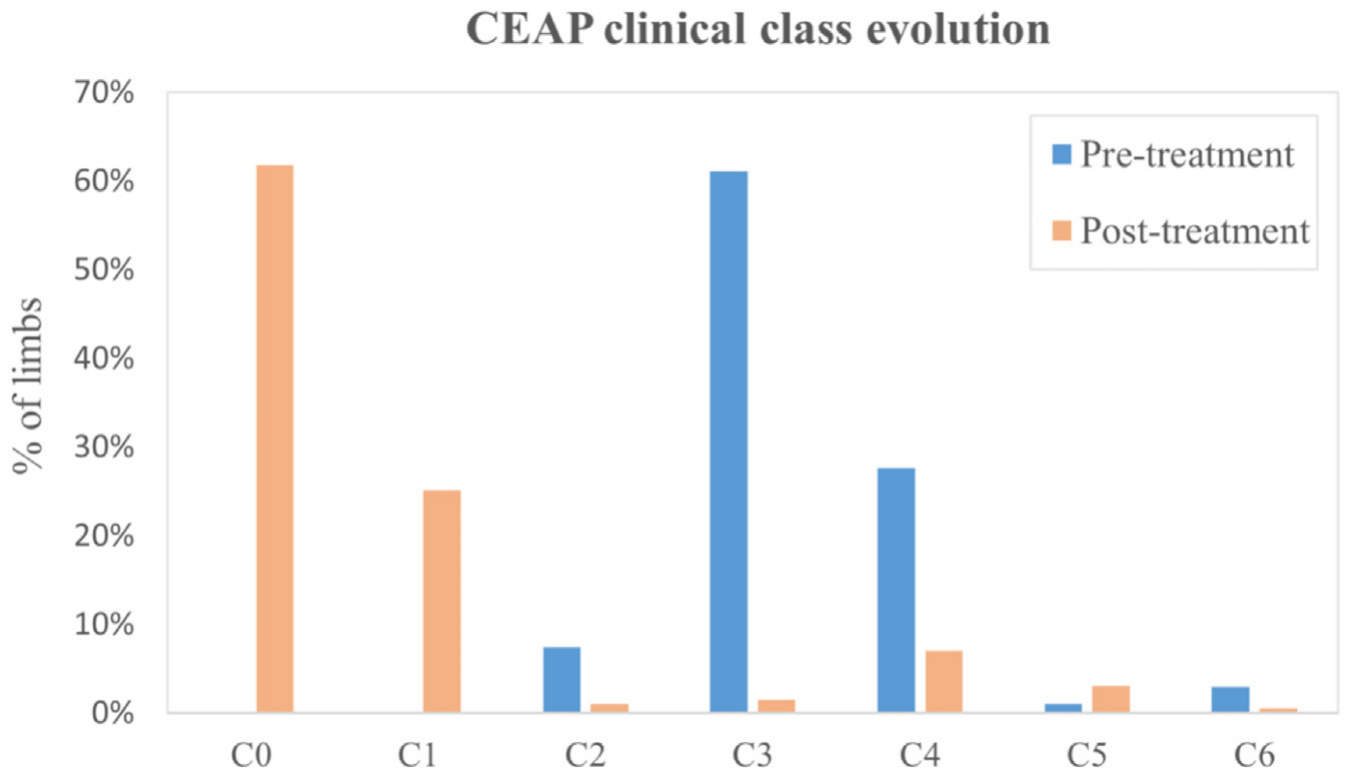
Clinical, Etiological, Anatomical, and Pathophysiological (CEAP) class distribution. *Pre*, pretreatment; *post*, post treatment.

After treatment, the mean rVCSS score was 1.3 (range, 0-10). Compared with the pre-surgery score, it improved significantly (*P* < .05). The clinical CEAP stages improved during the follow-up period. At the last evaluation, the score was class 0 in 123 (62.1%), class 1 in 49 (24.7%), class 2 in two (1%), class 3 in three (1.5%), class 4 in 14 (7.1%), class 5 in six (3%), and class 6 in one (0.5%). CEAP class improved significantly after the intervention (*P* < .0001) (Fig 8).

Among the six patients presenting with ulcers before the procedure, five were healed at the last follow-up visit.

No serious adverse events occurred in our series, and only a few minor complications were observed: one fat liquefaction (0.5%), three superficial vein thrombosis (1.6%), two skin bruising (1.1%), and two sural dysesthesias (1.1%) were reported.

## Discussion

Since its introduction in the late 1990s, EVTA has become the preferred treatment for venous insufficiency, largely replacing open surgery.^1^^,^^2^ Over the past 2 decades, numerous attempts have been made to develop alternative methods, primarily catheter-based, although with limited success. Venous tortuosity, small caliber, or short vein segments, especially in perforating veins, limit the use of catheter-based techniques. HIFU offers a versatile approach, capable of targeting and occluding specific venous segments, independent of vessel tortuosity, length, or caliber. Alfred Obermayer pioneered the clinical application of HIFU for CVD, presenting a case in 2018 at the VEITH symposium of a perforator vein.^6^ Inspired by this first historical case for local treatment, we explored the use of HIFU as an appropriate tool for implementing the CHIVA strategy. Indeed, for over 2 decades, CHIVA principles have been applied in our institution, replacing open surgery by EVLT, with excellent results but with the same anatomical and technical limitations mentioned above. This experience was reported in the PhD work of Zotta Desboeufs RV, in 2017.^13^ With the emergence of HIFU in the field of CVD, our approach was to consider it an appropriate tool for implementing the CHIVA strategy.

The endpoints of the study were vein occlusion, reflux abolition, and improving clinical and safety outcomes. The 24-month follow-up showed durable vein occlusion and elimination of venous symptoms. The HIFU thermal ablation outcomes are comparable to those obtained with EVLT and radiofrequency ablation.^2^^,^^14^^,^^15^ These results are consistent with those described in recent HIFU-related articles by Whiteley et al and Casoni et al, who reported their findings in the treatment of incompetent truncal/perforator veins and GSV respectively, at 12-month follow-up.^7^^,^^8^ By focusing on the source of leakage and selectively treating the impacted vein segments, the CHIVA-HIFU association appears to provide more accuracy in the control of reflux abolition, resulting in improved hemodynamic and clinical outcomes.

The vein closure may occur at the time of treatment, although it generally occurs progressively over the following weeks. As observed in our cohort, the closure rate at 1 month is higher than the closure rate at day 7. The closure is maintained for at least 24 months. In three limbs in which the leaking point remained patent, the reflux disappeared by the vein wall shrinking, restoring the competence of the ostial valve. This same event has been described by Casoni et al, but its clinical relevance is not yet known.^8^ The concomitant use of foam sclerotherapy may raise the question of whether the success rates are due solely to HIFU or also to the effects of the sclerosant. If so, the foam should completely thrombose the saphenous vein. However, subsequent ultrasound follow-up showed that these veins are remaining patent, with the exception of the HIFU-treated segment, ruling out the effect of sclerotherapy on the procedure outcome.

Our results also show a clinical improvement, with a statistically significant reduction in post-treatment rVCSS and improvement in post-treatment CEAP classification category, consistent with those reported with EVTA and the other HIFU experiences.^2^^,^^8^^,^^15^

In this series, the side-effect rates are minimal. This is particularly evident in this cohort, which covers the learning curve starting from the very first patients treated. The superficial thrombosis rate is remarkably low. This can be attributed to the systematic use of thromboprophylaxis as well as our conservative approach to the N3 treatment using low concentration polidocanol foam in small quantities, and in a staged strategy, with complementary ambulatory sclerosis during the follow-up if needed.

This underscores the excellent safety profile of the technology. HIFU minimizes the risk of adverse effects such as bleeding, infection, or thermal damage to surrounding tissues, including nerve injury. As pointed out by Casoni et al, the results suggest that the safety profile of HIFU is better than that of endovenous thermal ablation methods, but future studies should be conducted to demonstrate that.^2^^,^^8^^,^^14^^,^^15^

These results confirm HIFU as an effective alternative to conventional thermal ablation techniques. Because of its extracorporeal approach, the HIFU technique allows thermal venous closure in patients with challenging anatomic features, such as tortuous veins or small caliber vessels, that are difficult to perform with conventional methods. As HIFU avoids the need for tumescent anesthesia and eliminates percutaneous access, this feature improves patient quality of life and simplifies the procedure, making it well-suited to outpatient settings.

Certain limitations must be acknowledged. This was a single-arm, retrospective cohort study, and the lack of randomization may introduce selection bias. Future randomized controlled trials are needed to confirm these results and to further refine patient selection criteria and procedural techniques.

## Conclusions

HIFU combined with the CHIVA strategy is a safe, effective, and minimally invasive option for the treatment of CVD. The technique achieves durable vein occlusion and cumulative reflux elimination. Results compare favorably with EVTA and other HIFU studies and confirm the potential for its routine practice.

## Author Contributions

Conception and design: LI, TP, AA

Analysis and interpretation: LI, TR

Data collection: LI, TP, SM, JG, AA

Writing the article: LI, SM, JG

Critical revision of the article: LI, TP, SM, JG, AA

Final approval of the article: LI, TP, SM, JG, AA

Statistical analysis: LI, SM, JG

Obtained funding: Not applicable

Overall responsibility: LI

## Funding

None.

## Disclosures

None.
